# Morbidity from in-hospital complications is greater than treatment failure in patients with *Staphylococcus aureus* bacteraemia

**DOI:** 10.1186/s12879-018-3011-2

**Published:** 2018-03-05

**Authors:** Natasha E. Holmes, J. Owen Robinson, Sebastiaan J. van Hal, Wendy J. Munckhof, Eugene Athan, Tony M. Korman, Allen C. Cheng, John D. Turnidge, Paul D. R. Johnson, Benjamin P. Howden, VANESSA study group, on behalf of the Australasian Society for Infectious Diseases (ASID) Clinical Research Network (CRN), Natasha E. Holmes, Paul D. R. Johnson, Benjamin P. Howden, John D. Turnidge, J. Owen Robinson, Sebastian J. van Hal, Wendy J. Munckhof, Eugene Athan, Tony M. Korman, Allen C. Cheng

**Affiliations:** 1 Department of Infectious Diseases, Austin Health, Austin Centre for Infection Research, PO Box 5555, Heidelberg, VIC 3084 Australia; 2 Department of Microbiology and Immunology, University of Melbourne, Melbourne, VIC Australia; 3 Department of Microbiology and Infectious Diseases, PathWest Laboratory Medicine-WA, Royal Perth Hospital, 197 Wellington Street, Perth, WA 6000 Australia; 4 Australian Collaborating Centre for Enterococcus and Staphylococcus Species (ACCESS) Typing and Research, School of Biomedical Sciences, Curtin University, Perth, WA Australia; 5 Department of Microbiology and Infectious Diseases, Royal Prince Alfred Hospital, Missenden Road, Camperdown, NSW 2050 Australia; 6 Department of Medicine, University of Western Sydney, Sydney, NSW Australia; 7 Infection Management Services, Princess Alexandra Hospital, 199 Ipswich Road, Woolloongabba, QLD 4102 Australia; 8 Department of Medicine, University of Queensland, St Lucia, QLD Australia; 9 Department of Infectious Diseases, University Hospital Geelong, Barwon Health, Bellerine Street, Geelong, VIC 3220 Australia; 10 Department of Medicine, Deakin University, Geelong, VIC Australia; 11 Department of Medicine, University of Melbourne, Parkville, VIC Australia; 12 Department of Infectious Diseases, Monash Health, 246 Clayton Road, Clayton, VIC 3168 Australia; 13 Department of Medicine, Monash University, Clayton, VIC Australia; 14 Department of Infectious Diseases, Alfred Hospital, 55 Commercial Road, Prahran, VIC 3181 Australia; 15 Department of Epidemiology and Preventive Medicine, Monash University, Prahran, VIC Australia; 16 Australian Commission on Safety and Quality in Health Care, Level 5, 255 Elizabeth Street, Sydney, NSW 2000 Australia; 17 Department of Paediatrics, University of Adelaide, Adelaide, SA Australia; 18 Department of Microbiology, Monash University, Clayton, VIC Australia; 19 Microbiological Diagnostic Unit, Doherty Institute for Infection and Immunity, 792 Elizabeth Street, Melbourne, VIC 3000 Australia

**Keywords:** *Staphylococcus aureus*, Bacteraemia, Treatment failure, Complication, Mortality

## Abstract

**Background:**

Various studies have identified numerous factors associated with poor clinical outcomes in patients with *Staphylococcus aureus* bacteraemia (SAB). A new study was created to provide deeper insight into in-hospital complications and risk factors for treatment failure.

**Methods:**

Adult patients hospitalised with *Staphylococcus aureus* bacteraemia (SAB) were recruited prospectively into a multi-centre cohort. The primary outcome was treatment failure at 30 days (composite of all-cause mortality, persistent bacteraemia, or recurrent bacteraemia), and secondary measures included in-hospital complications and mortality at 6- and 12-months. Data were available for 222 patients recruited from February 2011 to December 2012.

**Results:**

Treatment failure at 30-days was recorded in 14.4% of patients (30-day mortality 9.5%). Multivariable analysis predictors of treatment failure included age > 70 years, Pitt bacteraemia score ≥ 2, CRP at onset of SAB > 250 mg/L, and persistent fevers after SAB onset; serum albumin at onset of SAB, receipt of appropriate empiric treatment, recent healthcare attendance, and performing echocardiography were protective. 6-month and 12-month mortality were 19.1% and 24.2% respectively. 45% experienced at least one in-hospital complication, including nephrotoxicity in 19.5%.

**Conclusions:**

This study demonstrates significant improvements in 30-day outcomes in SAB in Australia. However, we have identified important areas to improve outcomes from SAB, particularly reducing renal dysfunction and in-hospital treatment-related complications.

**Electronic supplementary material:**

The online version of this article (10.1186/s12879-018-3011-2) contains supplementary material, which is available to authorized users.

## Background

*Staphylococcus aureus* bacteraemia (SAB) remains an important and frequent cause of morbidity and mortality in hospitalised patients in spite of antimicrobial therapy, availability of supportive care, and improvements in hand hygiene and infection prevention. Much research has been undertaken in order to elucidate risk factors for mortality or treatment failure in patients with SAB, and these can be categorised broadly as factors related to the host, the pathogen, or antimicrobial treatment [1, 2]. Many of these studies have been retrospective in nature. We were interested in identifying risk factors associated with treatment failure and mortality of SAB, and describing in-hospital complications related to treatment and hospitalisation as well as long-term outcomes. A new prospective multi-centre cohort of patients with SAB was created to provide deeper insight into host and pathogen factors associated with treatment outcome and in-hospital complications.

## Methods

### Study design and population

The VANESSA (Vancomycin Efficacy in Staphylococcal Sepsis in Australia) cohort was established as a prospective observational study of patients with SAB in Australia. Eight urban tertiary referral hospitals recruited hospitalised adult patients (age ≥ 18 years) with at least one blood culture positive for SAB, including methicillin-susceptible *S. aureus* (MSSA) and methicillin-resistant *S. aureus* (MRSA), from 1 February 2011 to 31 December 2012. Potential participants were screened and approached by site investigators after SAB had been confirmed in the microbiology laboratory. Exclusion criteria included: 1) polymicrobial bacteraemia (including possible contaminants such as coagulase negative staphylococci, alpha-haemolytic streptococci or coryneform bacteria); 2) hospital admission to a non-study site or no hospitalisation; 3) transfer from another hospital with diagnosis of SAB but no positive blood culture at study site; 4) failure to provide informed consent. Repeat blood cultures were collected every 24-48 hours after diagnosis of SAB to document clearance of bacteraemia. Participants were followed for 12 months after the onset of SAB.

### Outcome measures

The 30-day composite endpoint of treatment failure comprised all-cause mortality, persistent bacteraemia (defined as positive blood cultures for seven or more days), or recurrent bacteraemia (defined as new positive blood cultures within 30 days of the index blood culture after documented clearance for 48 hours or more). Secondary outcomes included 30-day attributable mortality as defined by Lodise *et al*. (positive blood cultures for SAB at time of death, death occurred before resolution of symptoms and signs of SAB, death occurred within 14 days after onset of SAB without another explanation, autopsy findings indicated SAB as cause of death, death certificated indicated SAB as cause of death) [3], long-term mortality at 6-months and 12-months, and treatment complications (in-hospital and antibiotic-related).

### Data collection

Clinical data collection was performed on standardised case record forms and comprised demographics, risk factors for *S. aureus* acquisition, comorbidities [4], disease severity markers [5–7], clinical manifestations and laboratory parameters (refer Additional file 1). More than one clinical manifestation or site of metastatic infection was permitted per patient. Uncomplicated bacteraemia was defined as an absence of a defined infection syndrome (eg. severe sepsis including shock, endocarditis, pneumonia, osteoarticular, skin and soft tissue, deep abscess, meningitis or central nervous system, epidural abscess), or the presence of a removable focus such as a peripheral intravenous catheter with no other manifestations. Endocarditis was defined according to the modified Duke criteria [8]. Pre-defined in-hospital treatment complications were persistent renal impairment not returning to baseline within 30 days after onset of SAB, requirement for inpatient rehabilitation, persistent fevers for 7 or more days after onset of SAB, hospital-acquired infection other than SAB, venous thrombosis, metastatic infection beyond the primary site of infection, and pressure (decubitus) ulcers. Community-onset bacteraemia was defined as a positive blood culture collected immediately prior to or within 48 hours of hospital admission. Healthcare attendance was defined as hospital contact not requiring overnight admission (eg. day procedure unit, ambulatory care, day oncology, haemodialysis, Hospital in the Home). Acute renal failure was recorded at the onset of SAB according to the APACHE II score [5] and defined as urine output < 410 mL during the initial 24 hours after onset of SAB or serum creatinine > 132 umol/L and no prior known renal disease) [9]. Nephrotoxicity was defined as an increase in serum creatinine by 44 μmol/L or by more than 50% (whichever is greater) from baseline on at least two consecutive tests during the period of antibiotic treatment and up to 72 hours later [10]. Potential concomitant nephrotoxins were defined as previously [11]. Antibiotic treatment was defined as empiric (prior to susceptibility results) and definitive (predominant antibiotic used for the longest duration during the 30 days after the onset of SAB). Empiric therapy was deemed adequate if in vitro susceptibility was demonstrated for the antibiotic received. Combination therapy was defined as receipt of more than one antibiotic with anti-staphylococcal activity against the infecting organism for ≥ 24 hours. Formal infectious diseases (ID) consultation was recommended at all participating hospitals for management of patients with SAB.

### Microbiologic testing

The first positive (index) blood culture isolate from each patient was tested in a central laboratory, including species confirmation, Vitek®2 (bioMérieux, Marcy l’Etoile, France) susceptibility, vancomycin and oxacillin minimum inhibitory concentrations (MIC) using Etest® (bioMérieux, Marcy l’Etoile, France) and vancomycin MIC using broth microdilution (BMD) [12] including half-step dilutions (possible concentrations were 0.25, 0.375, 0.5, 0.75, 1, 1.5, 2, 3, 4, 6, 8, 12, 16, and 24 mg/L). Elevated vancomycin MIC was defined as ≥ 1.5 mg/L using Etest®.

### Statistical analysis

Statistical analysis was performed using Stata v11.2 (StataCorp, College Station, TX, USA). The chi-square test was used to compare categorical variables and the Mann-Whitney U test was used for continuous non-parametric variables. In order to generate binary variables from continuous data with wide ranges of plausible values, classification and regression tree (CART) analysis was performed to identify significant discriminatory values associated with the outcome of interest to generate a binary variable (Salford Predictive Modeler v7.0, Salford Systems, San Diego, CA, USA). Potentially significant variables on univariable analysis (*p*<0.2) were considered *a priori* for inclusion in a multivariable logistic regression model. Pairwise correlation coefficients were examined between variables that were potentially related before inclusion in our multivariable model to avoid collinearity, and by determining variance inflation factors during logistic regression. Stepwise backward elimination was performed and the Hosmer-Lemeshow statistic was performed to test the goodness of fit of the final model. A *p*-value of <0.05 was considered statistically significant.

## Results

### Description of cohort

226 patients were recruited into the cohort with complete data for 222 patients (refer Additional file 1). The population was predominantly male, Australian-born, and non-indigenous, with a median age of 62 years (interquartile range [IQR] 49-76) (Additional file 2: Table S1). Among the 51 patients (23.0%) who were admitted to the intensive care unit (ICU), the reason was directly related to SAB in 64% (32/50; 1 response not received). The main reasons for ICU transfer were haemodynamic support (10/51, 19.6%) and organ support for two or more systems (12/51, 23.5%).

### Patient exposures, comorbidities and disease severity

The most common potential exposures for acquisition of *S. aureus* prior to SAB onset included companion animal exposure, recent hospitalisation, recent healthcare attendance, and prior antibiotic therapy (Additional file 2: Table S1). Vancomycin accounted for almost one-quarter of antimicrobials used in those with prior antibiotic therapy (17/75, 22.7%; 2 responses not received). In patients where data were available, known prior colonisation with *S. aureus* was also a potential exposure (34/75, 45.3%). The most frequent comorbidities were cardiovascular disease, diabetes mellitus and chronic kidney disease (Additional file 2: Table S1). Dialysis was required in 25/48 (52.1%) patients with pre-existing end-stage renal disease, of which 14 (56.0%) were through a native arteriovenous fistula.

### Clinical and laboratory features

The most common clinical manifestations associated with SAB were: device-associated (81/222, 36.7%), osteoarticular (59/222, 26.6%), skin or soft tissue infection (46/222, 20.7%), uncomplicated bacteraemia (28/222, 12.6%), endocarditis (25/222, 11.3%), pneumonia (15/222, 6.8%), deep abscess (13/222, 5.9%), epidural abscess (10/222, 4.5%), severe sepsis including septic shock (7/222, 3.2%), central nervous system (1/222, 0.5%), and unknown foci (1/222, 0.5%); 22 patients (9.9%) had other manifestations not specified including intra-abdominal, urinary, septic thrombophlebitis, mediastinitis or ophthalmic foci. Echocardiography was performed in 91.9% (204/222) patients; the majority received transthoracic echocardiography alone (110/204, 53.9%). Endocarditis was mainly native valve (19/25, 76.0%) and left-sided (17/25, 68.0%). The most common device-associated infections were peripheral intravenous catheters (27/81, 33.3%), haemodialysis access (10/81, 12.3%), orthopaedic devices (10/81, 12.3%), and peripherally-inserted central venous catheters (7/81, 8.6%). Implanted devices were present in 69 patients (31.1%); 41/69 (59.4%) manifest as device-associated infection with SAB. In patients with device-associated infection, the device was removed in 75.3% (61/81) within a median of one day (IQR 0-2) of SAB onset.

Laboratory features included a median white cell count of 11.3 x10^9^/L (IQR 8.1-16.9), median C-reactive protein (CRP) of 170.5 mg/L (IQR 100-277.6) [reference range <10 mg/L] and median albumin of 30 g/L (IQR 26-34). Acute renal failure at the onset of SAB [9] was present in 22.1% (49/222).

### Microbiologic features

MRSA accounted for 25.7% (57/222) of all episodes of SAB. The median duration of bacteraemia was one day (IQR 1-3 days), with the longest duration of 13 days in a patient with recurrent bioprosthetic aortic valve endocarditis and a chronic forearm wound with failed split skin grafting. Elevated vancomycin MIC ≥ 1.5 mg/L was present in 39.2% (87/222) using Etest® and 32.9% (73/222) using BMD.

### Treatment

There was significant variability in antimicrobial treatment – antibiotics during a treatment course changed for a variety of reasons such as concurrent non-*S. aureus* infection requiring broader spectrum, toxicity, allergy or intolerance; thus the predominant definitive treatment received was flucloxacillin in 59.0% (131/222), vancomycin in 26.1% (58/222), and other antibiotics in 14.9% (33/222). Within the first seven days of therapy, the median flucloxacillin daily dose was 8 g (IQR 8-10.4 g) and the median vancomycin daily dose was 22.2 mg/kg (IQR 16.0-29.9 mg/kg). Nine patients with MSSA bacteraemia received vancomycin as the predominant antibiotic treatment; five of these had previous or new allergy or intolerance to β-lactams. Empiric treatment was adequate in 95.5% (212/222) of patients; in the 10 patients who received inappropriate empiric therapy, all had MRSA bacteraemia and received beta-lactams or aminoglycosides as initial therapy. In patients treated with definitive vancomycin, the median vancomycin trough concentration within 96 hours of treatment was 18 mg/L (IQR 14.2-22 mg/L).

One-third (74/222, 33.3%) of patients required surgical management as part of therapy; this included procedures to remove implanted devices such as prostheses and portacaths as well as aspiration, drainage or debridement.

### In-hospital complications

Forty-five percent (100/222) of patients experienced at least one pre-defined complication during hospitalisation: persistent renal impairment not returning to baseline within 30 days after onset of SAB (35/222, 15.8%), requirement for inpatient rehabilitation (35/222, 15.8%), hospital-acquired infection other than SAB (30/222, 13.5%), persistent fevers ≥ 7 days (24/222, 10.8%), metastatic infection beyond the primary site of infection (17/222, 7.7%), venous thrombosis (7/222, 3.2%), and pressure (decubitus) ulcer (3/222, 1.4%).

Adverse drug reactions (ADR) attributed to antimicrobial therapy occurred in 13.6% (30/221). These were attributed mainly to flucloxacillin (20/30, 66.7%) and vancomycin (7/30, 23.3%) but also included linezolid (1/30, 3.3%) and piperacillin-tazobactam (1/30, 3.3%); in two cases it was difficult to determine which antimicrobial was implicated, and one patient experienced ADR with two antibiotics at different time points during treatment. Of these ADRs, eight (8/30, 26.7%) were considered serious [13] such as sensorineural hearing loss (vancomycin), Stevens-Johnson syndrome (vancomycin), angioedema (flucloxacillin), and fevers requiring re-admission to hospital (vancomycin). Nephrotoxicity occurred in 19.5% (43/221) occurring in 14/58 (24.1%) patients receiving definitive vancomycin compared with definitive flucloxacillin (24/131, 18.5%) or other antimicrobials (5/33, 15.2%) (*p*=0.526). No patients classified with nephrotoxicity underwent renal biopsy to confirm antibiotic-induced kidney injury. Many patients (65.6%, 145/221) also received concomitant potential nephrotoxins regardless of kidney injury; these included Angiotensin Converting Enzyme Inhibitors (ACEIs) or Angiotensin Receptor Blockers (ARBs) (19/145, 13.1%), loop diuretics (17/145, 11.7%), radiocontrast dye (16/145, 11.0%), and non-steroidal anti-inflammatory drugs (NSAIDs) (14/145, 9.7%). Concomitant aminoglycosides (except those used in empiric treatment) were only prescribed in 1.4% (2/145). Concomitant nephrotoxins were prescribed more frequently in vancomycin-treated patients (40/57, 70.2%) in association with higher nephrotoxicity rates compared with flucloxacillin-treated patients (79/131, 60.3%).

### Clinical outcomes

The composite 30-day endpoint of persistent bacteraemia, recurrent bacteraemia or all-cause mortality was present in 14.4% (32/222). Thirty-day all-cause mortality was 9.5% (21/222), of which the majority were directly attributed to SAB (15/21, 71.4%). More than two thirds were still receiving anti-staphylococcal antibiotics (132/199, 66.3%) at 30 days. The median length of hospital stay after onset of SAB was 30 days (IQR 17-46 days); in some jurisdictions this included home parenteral antibiotic therapy. All-cause mortality at 6 months and 12 months was 19.1% (42/220) and 24.2% (53/219) respectively.

### Factors associated with 30-day treatment failure

Many variables were identified as potentially significant (*p*<0.2) for inclusion in the multivariable logistic regression model (Table 1). However with the low event rate, the number of candidate variables was restricted *post-hoc* to variables with *p*<0.1. Prior to model selection, we removed variables that were expected to be collinear or were directly related to outcome, eg. presence of DNR order is directly linked with mortality and was excluded. Age is part of APACHE II or SAPS II score however as these scores were validated in ICU populations, we elected to use age as the representative variable for inclusion. Patients with dementia are likely to reside in a long-term care facility, so we elected to use dementia rather than residence in a long-term care facility. Using backward stepwise elimination, variables that were associated with 30-day treatment failure were age > 70 years, Pitt bacteraemia score ≥ 2, CRP at onset of SAB > 250 mg/L, and persistent fevers after SAB onset. Elevated vancomycin MIC was not associated with treatment failure in this cohort. Higher serum albumin at onset of SAB, performing echocardiography, receipt of appropriate empiric therapy, and recent healthcare attendance prior to SAB onset were protective. Goodness of fit of the final model appeared to be satisfactory (Hosmer-Lemeshow statistic 4.82, *p*=0.777).

**Table 1:** Final multivariable logistic regression model of risk factors for treatment failure at 30 days in patients with SAB

Variable	Univariate analysis	Multivariable logistic regression		
	*p*-value	Odds ratio	95% CI	*p*-value
Age > 70 years	<0.001	4.13	1.54-11.07	0.005
Albumin (g/L)	<0.001	0.88	0.80-0.96	0.003
CRP > 250 mg/L	0.002	5.41	1.93-15.19	0.001
Persistent fevers	0.029	6.65	1.73-25.62	0.006
Healthcare attendance	0.003	0.13	0.03-0.59	0.008
Pitt bacteraemia score ≥ 2^a^	0.04	2.94	1.03-8.39	0.044
Echocardiogram performed	0.092	0.23	0.06-0.94	0.041
Appropriate empiric treatment	0.018	0.14	0.02-0.85	0.032
DNR order^b^	<0.001			-
WCC (x 10^9^/L)	0.048			-
ICU admission	0.003			-
APACHE II score > 11.5^b^	0.003			-
Combination treatment > 7 days	0.026			-
Severe sepsis	0.029			-
Body mass index (kg/m^2^)	0.037			-
Directed vancomycin treatment	0.044			-
Dementia	0.055			-
Directed flucloxacillin treatment	0.058			-
Nephrotoxicity	0.068			-
ARF at onset of SAB	0.07			-
Residence in LTCF^b^	0.09			-
Female sex	0.091			-
MRSA	0.098			-

^a^Score derived by CART analysis was > 1.5; as calculation of the score requires whole numbers, this is assumed to signify a Pitt bacteraemia score of ≥ 2

^b^Removed from multivariable model due to collinearity

*CRP* C-reactive protein, *DNR* do not resuscitate order, *WCC* white cell count, *ICU* intensive care unit, *ARF* acute renal failure, *SAB staphylococcus aureus* bacteraemia, *LTCF* long-term care facility, *MRSA* methicillin-resistant *S. aureus*, *CART* classification and regression tree, *CI* confidence interval

## Discussion

Results from this cohort have generated new insights about clinical features, treatment, and complications of SAB, and reinforced data from previous studies. The major findings from this study include lower than expected 30-day treatment failure and all-cause mortality, persistently high longer-term mortality, and morbidity from significant in-hospital treatment complications in patients with SAB in Australia. Whilst a number of strategies have undoubtedly impacted on short-term mortality rates, future research and interventions need to be directed at reducing other morbidity associated with treatment and hospitalisation for SAB.

Community-onset MSSA is still the predominant cause of SAB in Australia and tends to affect older males [14]. Among the multiple potential risk factors for acquisition of *S. aureus* analysed, the most frequent was animal exposure; however no sampling or testing of animal isolates was performed to determine any similarities between human-animal pairs. International travel has also been implicated in the spread of MRSA clones [15] however this was not a common risk factor for *S. aureus* acquisition in our cohort; this may be in part due to the presence of more MSSA in our study, and the relationship between travel and spread of MSSA clones is less well established. In addition, higher BMI was associated with treatment failure on univariate analysis, and obesity has been associated with an increased risk of colonisation with *S. aureus* [16], antibiotic treatment failure and mortality [17, 18].

In-hospital complications associated with SAB can be attributed to underlying host factors and complications related to the disease, hospitalisation, and treatment administered. Here we see a significant burden of persistent renal impairment after SAB, nosocomial infections, persistent fevers and vascular thrombosis during hospitalisation, and that almost one in six patients experienced an adverse event attributed to antimicrobial therapy. Although not statistically significant, nephrotoxicity was numerically more frequent in patients receiving definitive vancomycin compared with flucloxacillin, although concomitant nephrotoxins were also prescribed more frequently in vancomycin-treated patients. Whilst risk factors for vancomycin-associated nephrotoxicity have been established, such as higher steady state concentrations [11, 19], higher daily dosage [10], longer duration of exposure [19], and ACEI or ARBs [20], it is difficult to determine causality due to concomitant nephrotoxins and changing renal function and haemodynamics as a consequence of the infection itself [21]. Although infrequent, renal toxicity also occurs in anti-staphylococcal penicillins (ASP) [22, 23] and can occur early in therapy [24]; multiple studies have reported higher rates of adverse events including acute kidney injury in patients receiving ASP compared with cefazolin [25–29]. However little is known about predictors of nephrotoxicity in flucloxacillin-treated patients, and this should be a focus of future research. Venous thrombosis is a recognised common complication of short- and long-term central venous catheter-related SAB [30], and it is possible that the effects of *S. aureus* surface proteins and exotoxins on the coagulation pathway may also contribute to vascular thrombosis in the absence of venous catheters [31].

An unexpected finding was the low rate of 30-day treatment failure (14.4%) in the cohort, predominantly driven by very low all-cause mortality (9.5%). Recent but not contemporaneous studies in Australia and New Zealand reported 30-day mortality rates of 17-20% [1, 32, 33] and although mortality rates have been reported as low as 6.8-13% in studies from Finland, Iceland and Taiwan, the vast majority of patients in these cohorts had MSSA bacteraemia [34–36]. In a pooled analysis of over 3300 patients from 5 large multi-centre international studies [14] 30-day mortality was 21% - much higher than the current study. There may be multiple reasons for this. First, identification of an infective focus was determined in 99.5% of our patients; the absence of an identified focus has been strongly associated with mortality [14]. Second, more than 95% of our patients received adequate empiric therapy, ensuring appropriate coverage in the first few critical days of therapy, and serum vancomycin levels were therapeutic early in therapy in those treated with vancomycin. Third, there was attention to focus eradication and source control as this is crucial in SAB management [37]; more than one-third of our patients required formal surgical management, three-quarters had their device removed within a median of 24 hours after the onset of SAB, and more than 91% underwent echocardiography. Fourth, there were fewer isolates with elevated vancomycin MIC (compared with our previous work [1]) which has been associated with treatment failure and mortality [38]. This may be due to different *S. aureus* genotypes in this cohort, and typing is underway. Fifth, mortality rates in critically ill patients with severe sepsis in Australia and New Zealand have fallen from 2000 to 2012 [39]; this secular trend is contemporaneous with our current study and suggests that supportive care and management of these patients have improved over this time and led to the favourable clinical outcomes found in our cohort. Although we did not specifically record formal ID consultation, all the participating hospitals had guidelines for ID consultation in patients with SAB. Furthermore 30-day mortality rates in patients with SAB have also fallen in our region from 20.6% in 2007-2008 [32] to 14.4% in 2013 [40]. Sixth, bias may have been introduced as time dependent variables will have changed the number of patients at risk for the outcome assessment and are not captured in a logistic regression analysis. Finally, there may have been an unintended bias from our enrolment strategy towards less critically ill patients or towards patients where treatment success was more likely. As informed consent was required to participate it was not appropriate to consent patients where death was imminent; some patients with severe disease and early death from or with SAB were therefore not included.

All-cause mortality continues to increase in patients following an episode of SAB, and this has also been noted in the literature [41–43]. Although we did not determine attributable mortality at 6-months and 12-months, the increased mortality seen is likely to reflect patient age and comorbidities as well as the consequences of complications that occur after an episode of SAB; our study demonstrates that in-hospital complications are frequent and cause morbidity that persist beyond 30 days and may contribute to overall increased mortality in the medium term.

Elevated vancomycin MIC was detected in approximately one-third of our isolates, yet interestingly there was no association with increased mortality or treatment failure. We have previously observed inferior clinical outcomes such as mortality or complicated infection in patients whose isolates have an elevated vancomycin MIC [1], and others have also reported similar findings even among patients with MSSA bacteraemia [44–46]. However the association between vancomycin MIC and poor clinical outcomes has been contentious. In the systematic review and meta-analysis performed by van Hal *et al.* [38] elevated vancomycin MIC was associated with a higher mortality rate in patients with MRSA infections, regardless of the source of infection or MIC methodology. In contrast Kalil *et al*. [47] found no statistically significant difference in mortality in patients with SAB. Even when assessing vancomycin MIC by the Microscan method, there was no difference in 90-day mortality, readmission or recurrence in a prospective single centre cohort [48]. Moreover there are also problems with the definition of elevated vancomycin MIC and differences in MIC methodology, so currently there are limited data to support changing clinical practice as a result of vancomycin MIC testing in order to improve clinical outcomes [49–51].

Predictors associated with treatment failure in this study, notwithstanding the low event rate of 30-day treatment failure, comprised clinical and biochemical variables that are easily obtained during the initial evaluation of a patient with SAB such as age, Pitt bacteraemia score, serum albumin and CRP. Significant elevations in CRP have also been associated with mortality in bacteraemic patients [52] and treatment failure in MRSA bacteraemia [53]. Recent healthcare exposure was associated with lower treatment failure; possible explanations may include higher incidence of uncomplicated line-associated bacteraemia, earlier hospital presentation when unwell, or earlier appropriate MRSA therapy in patients known to be colonised. Persistent fevers and performing echocardiography were also predictors of treatment response and this is presumably related to underlying source identification and focus eradication as important adjuncts in SAB management. Appropriate empiric therapy was also associated with lower treatment failure, consistent with the literature that delays in appropriate therapy are associated with inferior outcomes [54, 55], although confounding by indication has also been observed when evaluating appropriate antibiotic therapy [56]. The wide confidence intervals for the odds ratios in the multivariable analysis reflect the low event rate of treatment failure and are another potential limitation of the study.

## Conclusions

In conclusion, we have documented a significant shift in the 30-day outcomes in patients with SAB across Australia, however in-hospital complications and longer-term mortality remain a significant burden that should be addressed in future studies of SAB.

## Additional files

Additional file 1: Appendix Source of clinical data, clinical covariates collected, study flowchart. (DOCX 174 kb)

Additional file 2: **Table S1.** Clinical characteristics and risk factors for *S. aureus* acquisition in cohort. (DOC 49 kb)

## Abbreviations

ACEI: Angiotensin converting enzyme inhibitor
ADR: Adverse drug reaction
ARB: Angiotensin receptor blocker
ASP: Anti-staphylococcal penicillin
BMD: Broth microdilution
CART: Classification and regression tree
CRP: C-reactive protein
ICU: Intensive care unit
ID: Infectious diseases
IQR: Interquartile range
MIC: Minimum inhibitory concentration
MRSA: Methicillin-resistant *Staphylococcus aureus*
MSSA: Methicillin-susceptible *Staphylococcus aureus*
NSAID: Non-steroidal anti-inflammatory drug
SAB: *Staphylococcus aureus* bacteraemia
VANESSA: Vancomycin efficacy in staphylococcal sepsis in Australia
