# Supplementary material for: Morbidity from in-hospital complications is greater than treatment failure in patients with Staphylococcus aureus bacteraemia
Source: BMC Infect Dis. 2018 Mar 5;18:107. doi: 10.1186/s12879-018-3011-2 (PMC5838938; doi:10.1186/s12879-018-3011-2)
Supplement: Supplementary file 1 — Appendix Source of clinical data, clinical covariates collected, study flowchart. (DOCX 174 kb) [file 12879_2018_3011_MOESM1_ESM.docx]

**Supplementary Appendix**

1. Primary sources of clinical data

- Patient
- Medical staff from the primary treating unit
- Medical record

1. Clinical covariates collected
2. *Potential exposures for* S. aureus *acquisition (within 30 days prior to onset of SAB):*

- Recent hospitalisation
- Recent surgery, including day surgery
- Recent healthcare or hospital exposure without overnight admission (eg. day procedure, ambulatory care, hospital in the home, day chemotherapy, haemodialysis)
- Resident in long-term care facility
- Inmate in correctional facility
- Injecting drug use
- Contact sports
- Animal exposure (companion, occupational, or both)
- Known colonisation with *S. aureus*
- Prior receipt of antibiotic therapy

1. *Comorbidities:*

- Charlson comorbidity score [1]
- Chronic skin disease
- Pressure (decubitus) ulcer
- Transplant (haematologic stem cell, solid organ, or both)

1. *Receipt of immunosuppression (one or more of the following):*

- Corticosteroids equivalent to prednisolone 20 mg daily or greater
- Tumour necrosis factor inhibitors
- Monoclonal antibodies (any)
- Cytotoxic chemotherapy
- Other immunosuppressants such as methotrexate, cyclosporin and calcineurin inhibitors

1. *Disease severity markers:*

- Acute Physiology And Chronic Evaluation (APACHE) II [2]
- Sepsis-related Organ Failure Assessment (SOFA) [3]
- Pitt bacteraemia score [4]

1. *Clinical manifestations of SAB:*

- Uncomplicated bacteraemia (no evidence of metastatic infection or end-organ involvement)
- Severe sepsis, including shock
- Endocarditis, left- or right-sided, native or prosthetic
- Pneumonia
- Osteoarticular, including discitis
- Skin and skin structure, including wound
- Deep abscess (eg. psoas)
- Epidural abscess
- Meningitis or central nervous system
- Device-associated (including removeable and non-removeable implanted devices)
- Other (not specified above)
- Unknown

1. *Potential concomitant nephrotoxins [5]:*

- Amphotericin B
- Radiocontrast dye
- Calcineurin inhibitors
- Loop diuretics
- Angiotensin converting enzyme inhibitors (ACEIs)
- Angiotensin receptor blockers (ARBs)
- Non-steroidal anti-inflammatory drugs (NSAIDs)
- Aminoglycosides

1. Study participation flowchart

**References**

1. Lesens O, Methlin C, Hansmann Y, et al. Role of comorbidity in mortality related to *Staphylococcus aureus* bacteremia: a prospective study using the Charlson weighted index of comorbidity. Infect Control Hosp Epidemiol **2003**; 24(12): 890-6.

2. Knaus WA, Draper EA, Wagner DP, Zimmerman JE. APACHE II: A severity of disease classification system. Crit Care Med **1985**; 13(10): 818-29.

3. Vincent JL, Moreno R, Takala J, et al. The SOFA (Sepsis-related Organ Failure Assessment) score to describe organ dysfunction/failure. On behalf of the Working Group on Sepsis-Related Problems of the European Society of Intensive Care Medicine. Intensive Care Med **1996**; 22(7): 707-10.

4. Paterson DL, Ko WC, Von Gottberg A, et al. International prospective study of *Klebsiella pneumoniae* bacteremia: implications of extended-spectrum beta-lactamase production in nosocomial Infections. Ann Intern Med **2004**; 140(1): 26-32.

5. Ingram PR, Lye DC, Tambyah PA, Goh WP, Tam VH, Fisher DA. Risk factors for nephrotoxicity associated with continuous vancomycin infusion in outpatient parenteral antibiotic therapy. J Antimicrob Chemother **2008**; 62(1): 168-71.
