# Supplementary material for: Morbidity from in-hospital complications is greater than treatment failure in patients with Staphylococcus aureus bacteraemia
Source: BMC Infect Dis. 2018 Mar 5;18:107. doi: 10.1186/s12879-018-3011-2 (PMC5838938; doi:10.1186/s12879-018-3011-2)
Supplement: Supplementary file 2 — Table S1. Clinical characteristics and risk factors for S. aureus acquisition in cohort. (DOC 49 kb) [file 12879_2018_3011_MOESM2_ESM.doc]

**Supplementary Table 1.** Clinical characteristics and risk factors for *S. aureus* acquisition in cohort (n=222).

| **Variable** | **Number (percentage)^1^** |
| --- | --- |
| *Demographics* | |
| Age, years (median, interquartile range [IQR]) | 62 (49-76) |
| Body mass index, kg/m^2^ (median, IQR) | 26.9 (23.5-31.0) |
| Male sex | 147 (66.2) |
| Australian-born | 152 (68.5) |
| Indigenous origin | 9 (4.0) |
| Penicillin allergy | 20 (9.0) |
| *Details of hospitalisation* | |
| Hospital onset | 75 (33.8) |
| Admission under medical unit | 173 (77.9) |
| Intensive care unit (ICU) admission | 51 (23.0) |
| Do Not Resuscitate (DNR) order | 32 (14.4) |
| Length of stay after onset of SAB^2^ (median, IQR) | 30 (17-46) |
| *Risk factors for acquisition^3^* | |
| Animal exposure | 89/218 (40.8) |
| Recent hospitalisation | 87 (39.2) |
| Prior antibiotic therapy | 77/220 (35.0) |
| Recent healthcare attendance | 70/221 (31.7) |
| Recent surgical procedure | 47/221 (21.3) |
| Receipt of immunosuppression | 44 (19.8) |
| Injecting drug use | 19/221 (8.6) |
| Chronic skin disease | 19/221 (8.6) |
| Residence in long-term care facility | 9/220 (4.1) |
| International travel | 5/221 (2.3) |
| Contact sports | 4/221 (1.8) |
| *Comorbidities and disease severity scores* | |
| Cardiovascular disease | 60 (27.0) |
| Diabetes mellitus | 53 (23.9) |
| Chronic kidney disease | 48 (21.6) |
| Solid organ tumour | 34 (15.3) |
| Chronic pulmonary disease | 31 (14.0) |
| Chronic liver disease | 25 (11.3) |
| Charlson score (median, IQR) | 2 (1-4) |
| APACHE II score (median, IQR) | 13 (8-18) |
| SOFA score (median, IQR) | 2 (0-4) |
| Pitt bacteraemia score (median, IQR) | 1 (0-2) |

IQR, interquartile range.

^1^ Except where indicated.

^2^ Including outpatient parenteral antibiotic therapy at institutions where this was considered part of the same episode of care.

^3^ Within 30 days prior to onset of SAB.
